# ZO-2 seals the deal for platelet tight junctions

**DOI:** 10.1016/j.rpth.2025.102952

**Published:** 2025-06-23

**Authors:** Yiheng Zhang, Owen J.T. McCarty, Joseph E. Aslan

**Affiliations:** 1 Department of Biomedical Engineering, Oregon Health & Science University, Portland, Oregon, USA; 2 Department of Chemical Physiology and Biochemistry, Oregon Health & Science University, Portland, Oregon, USA; 3 Knight Cardiovascular Institute, Oregon Health & Science University, Portland, Oregon, USA

Platelet adhesion and aggregation are essential for hemostatic plug and thrombus formation [1]. While molecular mechanisms underlying platelet–platelet cross-bridging and aggregate formation through the activation of integrins are actively and extensively studied [2], several questions remain about how platelets establish and maintain physically stable and dynamic contacts with one another. Platelets are increasingly recognized for regulating their defining adhesion, secretion, and aggregation responses through specific cytoskeletal processes [3,4]. Each of these plays an important role in regulating thrombus architecture, mechanical integrity, and stability under conditions of blood flow [5].

In addition to cell adhesion molecules such as integrins and selectins, tight junction (TJ) proteins also mediate cell–cell adhesion and barrier formation in epithelial tissues [6,7]. Interestingly, some TJ-associated proteins, including ESAM [8] and F11R/JAM-A [9], have been identified and characterized for specific roles in platelet function. Proteomics studies have also noted that many other junction proteins are highly expressed in platelets and remain unstudied, including zonula occludens 2 (ZO-2), also known as TJ protein TJP2 [10,11]. Whether or not these components work together to establish functional TJs in platelets and influence thrombus architecture has not been directly addressed.

In this issue of *Research and Practice in Thrombosis and Haemostasis*, Nagy et al. [12] detail the roles of ZO-2 in establishing TJ-like structures at platelet–platelet contact sites. Using a combination of platinum replica electron microscopy, superresolution dSTORM imaging, and confocal microscopy, this study provides striking, high-resolution visual and physiologic evidence for the presence and function of TJ-like structures at interplatelet contact sites.

The authors first imaged an intermingling of the platelet actin cytoskeleton and putative TJ structures with platinum replica electron microscopy. Using fluorescence confocal and superresolution microscopy, Nagy et al. [12] then examined the dynamics of TJ proteins in platelets – especially ZO-2 – to gain insights into their potential function. After treating adherent human platelets with a variety of agonists, ZO-2 was redistributed to distinct, elongated clusters at sites of contact with neighboring platelets. Superresolution microscopy further revealed that these clusters consist of parallel strings of ZO-2 molecules, symmetrically aligned across adjacent platelets, forming an interdigitated structure reminiscent of classical TJs. Importantly, the formation of these structures was dynamically regulated by actin polymerization and depolymerization, as well as integrin activation. Moreover, while prior phosphoproteomics studies detected extensive phosphorylation of ZO-2 in activating platelets [13], treatment of platelets with the protein kinase A activator iloprost prevented ZO-2 redistribution to TJ-like clusters.

Finally, to directly examine the roles of TJ-like contacts in platelets, the authors took advantage of a blood sample from a patient with a rare genetic deficiency in ZO-2. Although ZO-2–deficient platelets from this subject did not cluster other junctional proteins, including ESAM and JAM-A, at sites of platelet–platelet contacts, thrombi formed by these platelets were markedly more stable over time and resistant to dissolution. These findings suggest that junction-like structures formed around ZO-2 play a role in thrombus remodeling and plasticity; as thrombi appear more stable in the absence of ZO-2, ZO-2–containing junctions may regulate a balance between thrombus formation and dissolution.

Altogether, this study from the Baaten group places the TJ protein ZO-2 as a functionally relevant player in cell–cell contacts between platelets (Figure). While integrin α_IIb_β_3_ activation initiates platelet aggregation [14], ZO-2 junction-like structures may refine the spatial organization of platelet aggregates and facilitate dynamic remodeling within thrombi. As activating platelets release an array of bioactive molecules, narrow gaps between closely apposed platelets create protected microenvironments where secreted factors may accumulate at high local concentrations, contributing to thrombus stabilization by enhancing fibrin cross-linking, modulating fibrinolysis, and reinforcing intercellular cohesion [15]. Given that thrombus stability critically influences outcomes in cardiovascular disease [16], stroke [17], abdominal aortic aneurysm [18], and other contexts, future studies of TJ-like structures represent an intriguing direction for future research in platelet physiology as well as hemostatic and thrombotic disorders.

**Figure:**
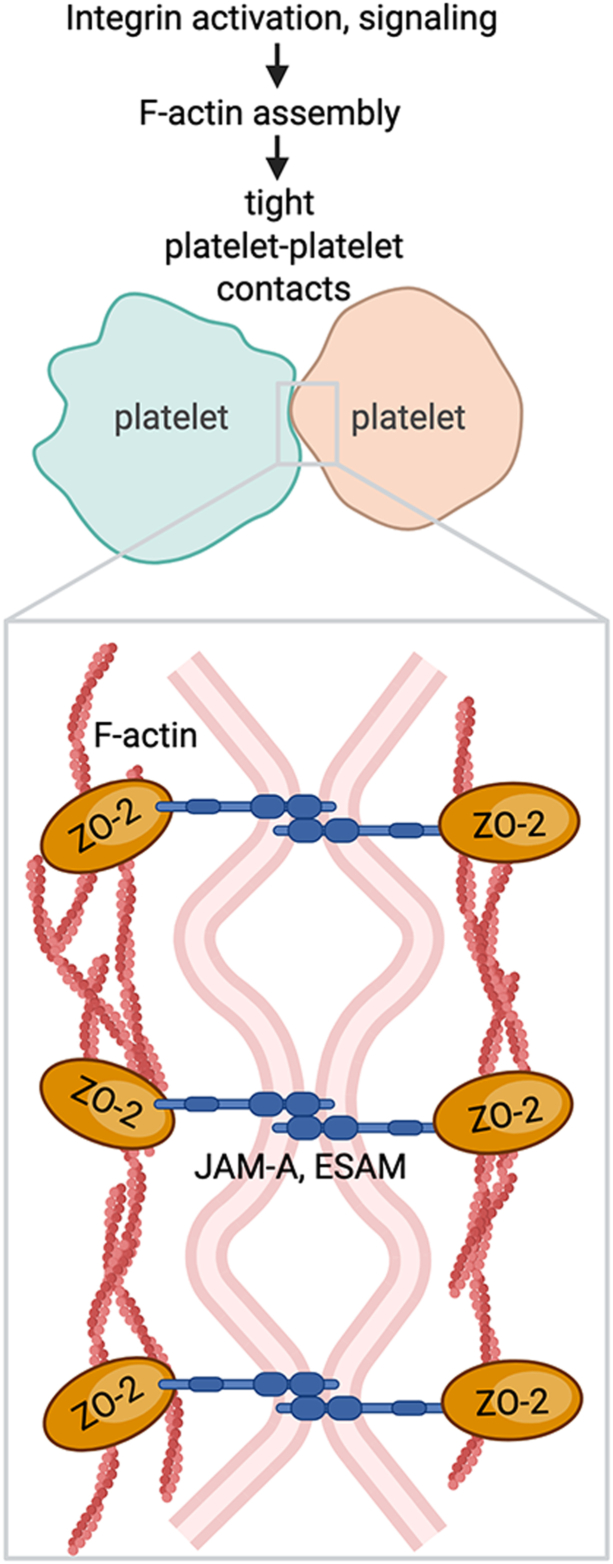
Nagy et al. [12] detail a role for zonula occludens 2 (ZO-2) proximal to integrin activation, filamentous F-actin assembly, and junctional protein clustering (eg, ESAM and JAM-A) in the formation of tight junction-like contacts between platelets.
